# Comparative chloroplast genomics of Rutaceae: structural divergence, adaptive evolution, and phylogenomic implications

**DOI:** 10.3389/fpls.2025.1675536

**Published:** 2025-10-03

**Authors:** Yichao Chen, Ningge Liu, Hong Wang, Huifeng Luo, Zhiliang Xie, Hongao Yu, Yaojun Chang, Bosheng Zheng, Xinchen Zheng, Jun Sheng, Yajie Jiang, Shuzhe Ye, Yonggang Hua, Haijie Ma, Fei Li

**Affiliations:** ^1^ Wenzhou Vocational College of Science and Technology, Wenzhou, China; ^2^ Key Laboratory of Quality and Safety Control for Subtropical Fruit and Vegetable, Ministry of Agriculture and Rural Affairs, Collaborative Innovation Center for Efficient and Green Production of Agriculture in Mountainous Areas of Zhejiang Province, College of Horticulture Science, Zhejiang A&F University, Hangzhou, Zhejiang, China; ^3^ Institute of Horticulture, Hangzhou Academy of Agricultural Sciences, Hangzhou, China; ^4^ Hangzhou Agricultural Technology Extension Center, Hangzhou, Zhejiang, China

**Keywords:** chloroplast genome, *Murraya paniculata*, Rutaceae, comparative genomics, adaptive evolution, phylogeny, selection pressure

## Abstract

The study of chloroplast genome evolutionary dynamics provides critical insights into plant adaptive evolution and phylogenetic relationships. This research conducted a systematic comparative analysis of chloroplast genomes across 35 species within the Rutaceae family. All genomes displayed the typical quadripartite structure, with sizes ranging from 155 to 161 kb, GC contents between 38.17% and 38.83%, and gene counts varying from 122 to 144. Structural conservation was high across species, with variations mainly localized at the boundaries of inverted repeat (IR) regions. AT-rich mononucleotide simple sequence repeats (SSRs) were dominant and primarily distributed in non-coding regions. Collinearity analysis revealed high sequence conservation alongside lineage-specific rearrangements. Relative synonymous codon usage (RSCU) analysis revealed significant heterogeneity among species, with values ranging from 0.386 to 1.797. ENC-GC3s, GC3-GC12, and PR2 analyses indicated a marked deviation from neutral evolution. Selection pressure analysis indicated strong purifying selection (Ka/Ks < 0.2) acting on photosynthetic system genes, while certain genes (e.g., *matK*, *rpl20*) exhibited signals of positive selection, highlighting adaptive evolutionary features in specific genomic regions. Phylogenetic reconstruction placed *Murraya paniculata* within a clade containing other *Murraya* species, closely related to *Citrus* and *Clausena*, reflecting morphological and biogeographic patterns. This study provides a molecular framework for taxonomic revision in Rutaceae and enhances understanding of chloroplast genome evolution in the family.

## 1 Introduction

Chloroplasts, as essential organelles for photosynthesis and various metabolic pathways in plants, have become a vital tool for phylogenetic reconstruction, species identification, and evolutionary studies due to their maternal inheritance, structural conservation, and moderate evolutionary rate (Daniell et al., 2016, 2021). The typical chloroplast genome exhibits a circular quadripartite structure, comprising a large single-copy region (LSC), a small single-copy region (SSC), and a pair of inverted repeats (IRs). It encodes approximately 110 – 150 genes, primarily involved in photosynthesis, transcription, translation, and metabolic regulation (Wicke et al., 2011; Jin and Daniell, 2015). Recent advancements in high-throughput sequencing technologies have significantly propelled research on chloroplast genomes, yielding remarkable progress in understanding plant phylogenetics and adaptive evolution (Ahmad and Nixon, 2025; Cauz-Santos, 2025; Li et al., 2015; Narra et al., 2025; Zhang et al., 2025; Zheng et al., 2025). However, comparative studies of chloroplast genomes within the Rutaceae family remain relatively limited, particularly for the genus *Murraya* and its closely related species. The lack of systematic characterization of their chloroplast genomic features and evolutionary mechanisms hinders a comprehensive understanding of the evolutionary history and adaptive strategies within this lineage.

Owing to their structural conservation, chloroplast genomes offer unique advantages for comparative genomics and phylogenetic studies. Systematic comparisons of genome size, IR boundary shifts, gene arrangement, simple sequence repeats (SSRs), codon usage bias, and protein-coding sequences can reveal genetic divergence and evolutionary trajectories among species (Agnello et al., 2016). SSRs, as highly variable elements predominantly located in non-coding regions, exhibit substantial polymorphism and serve as valuable tools for analyzing interspecific relationships and developing molecular markers (Ping et al., 2021; Zhao et al., 2022). In eukaryotes, 61 codons encode 20 amino acids, and codon usage bias is shaped by a combination of evolutionary forces, including natural selection, mutational bias, and genetic drift (Quax et al., 2015; Parvathy et al., 2022). Studies have demonstrated that codon usage preferences in chloroplast genomes are often correlated with gene expression levels, translational efficiency, and functional importance (Frumkin et al., 2018; Yang et al., 2023a). Such biases are generally regarded as the outcome of a balance between non-synonymous codon mutation drift and selective pressures favoring optimal codons (Behura and Severson, 2013). Additionally, these biases are influenced by multiple factors, including nucleotide composition, GC content, gene expression levels, and tRNA abundance (Romero et al., 2000; Blake et al., 2003; Schwark et al., 2020; Niu et al., 2021). For instance, highly expressed photosynthesis-related genes often exhibit stronger codon preferences, a phenomenon attributed to natural selection optimizing translational efficiency (Yang et al., 2023b). Furthermore, the ratio of non-synonymous to synonymous substitution rates (Ka/Ks) can identify functional genes under positive selection, providing insights into the role of specific genes in adaptive evolution. Multidimensional comparative analyses not only elucidate the conservation and variability of chloroplast genomes but also offer theoretical foundations for species identification, taxonomic revision, molecular adaptation to environmental changes, gene expression regulation, and resource conservation (Tang et al., 2000; Zhou et al., 2016).

Collinearity analysis and phylogenetic reconstruction based on chloroplast genomes are pivotal methods for investigating plant evolutionary history (Jansen et al., 2007; Li et al., 2019). Compared to traditional single-gene or multi-gene fragment approaches, whole-genome sequences provide more comprehensive phylogenetic signals, particularly in resolving complex relationships among closely related species (Lemmon and Lemmon, 2013; Wickett et al., 2014). Within Rutaceae, previous studies have predominantly focused on economically significant groups such as *Citrus*, while the systematic positions of genera like *Murraya* remain understudied (Carbonell-Caballero et al., 2015). Moreover, Ka/Ks analysis can delineate evolutionary patterns of different genes and identify functional genes potentially undergoing adaptive evolution (Hurst, 2002). These analytical approaches will provide novel insights into the evolutionary history and adaptive mechanisms of *Murraya* species. In recent years, the growing accumulation of chloroplast genome sequences in public databases has enabled systematic comparative studies at both genus and family levels (Li et al., 2025a, 2025). For taxonomically ambiguous or contentious groups, leveraging existing chloroplast data with well-designed sampling strategies has become a key trend in molecular phylogenetic research.

This study systematically investigates the chloroplast genomes of 35 Rutaceae species, focusing on structural characteristics, sequence variation, repeat sequences, codon usage bias, collinearity, selection pressure, and phylogenetic relationships. The research aims to elucidate the conservation and variability of chloroplast genomes within Rutaceae and explore the genetic basis underlying phylogenetic divergence and ecological adaptation. Notably, this study reports the complete chloroplast genome of an important *Murraya* germplasm resource, integrating it into a family-wide comparative framework to clarify its phylogenetic position and relationships within Rutaceae. The findings not only provide molecular evidence for resolving complex taxonomic issues in Rutaceae but also offer new perspectives and theoretical support for understanding the adaptive evolution and conservation of biodiversity within this family.

## 2 Materials and methods

### 2.1 Plant material collection and DNA sequencing

Fresh leaves of *Murraya paniculata* were collected from Fuzhou city, Fujian Province, China. Plants were cultivated under controlled greenhouse conditions (temperature: 24 - 26 °C; humidity: 50 - 70%; photoperiod: 16 h light/8 h dark). Genomic DNA was extracted from fresh leaf tissue using an improved CTAB method (Liu et al., 2025). High-throughput sequencing was performed on the DNBSEQ-T7 platform (MGI Tech), generating approximately 20 GB of 150 bp paired-end raw reads per sample, achieving approximately 100× coverage depth of the chloroplast genome. The complete chloroplast genome sequence of *M. paniculata* has been deposited in the NCBI GenBank database under accession number PX214363. For comparative analysis, chloroplast genome sequences of 34 additional Rutaceae species were retrieved from the NCBI database (https://www.ncbi.nlm.nih.gov/).

### 2.2 Chloroplast genome assembly and annotation

The complete chloroplast genome of *Murraya paniculata* was assembled using assembled using oatk (Organellar Assembly Toolkit) with PacBio HiFi sequencing data. The assembly was performed using the following parameters: k-mer size of 1001 (-k 1001), coverage threshold of 150 (-c 150), and 8 threads (-t 8). Organellar genome identification and separation was achieved using angiosperm-specific HMM profiles for mitochondrial genomes. Assembly quality was rigorously validated using QUAST v5.0.2, revealing high-quality metrics with N50 values ranging from 87,592 bp (LSC region) to 26,994 bp (IR regions), total assembly length of 160,179 bp, and zero assembly gaps. Preliminary genome annotation was conducted using the GeSeq tool with default settings (Tillich et al., 2017), referencing annotated chloroplast genomes from closely related *Murraya* species to enhance accuracy. To further ensure the precision of gene identification, particularly regarding start/stop codons and exon-intron junctions, all annotations were manually curated and refined using the Sequin software package provided by NCBI. A circular chloroplast genome map was then generated using the web-based visualization tool Chloroplot (https://irscope.shinyapps.io/Chloroplot/). This map illustrates the typical quadripartite structure of the genome and shows the precise locations and orientations of genes across the large single-copy (LSC), small single-copy (SSC), and inverted repeat (IR) regions.

### 2.3 SSR analysis

Simple sequence repeat (SSR) analysis was conducted to characterize the distribution and composition of microsatellites across the chloroplast genomes. SSRs were identified using IMEx v2.1 software (Mudunuri and Nagarajaram, 2007) with the following minimum repeat thresholds: 10 repeats for mononucleotides, 5 for dinucleotides, 4 for trinucleotides, and 3 for tetra- to decanucleotides. In this study, repeats of >6 bp were included to provide a more comprehensive survey of chloroplast SSR diversity. Although SSRs are typically defined as 1 – 6 bp units, larger motifs occur at lower frequency and can contribute to species-specific polymorphism and comparative resolution. Detected SSRs were systematically classified based on: (1) repeat unit length (mono- to decanucleotide), (2) genomic location (coding regions, introns, or intergenic spacers), and (3) nucleotide composition (AT-rich or GC-rich motifs). The distribution patterns of SSR types were visualized as a heatmap using the R package *pheatmap*, highlighting interspecific variations in SSR abundance and composition. This analysis revealed the prevalence of AT-rich SSRs, particularly in noncoding regions, reflecting mutation biases and selective constraints in chloroplast genome evolution.

### 2.4 RSCU analysis

The Relative Synonymous Codon Usage (RSCU) method was employed to evaluate codon usage bias across chloroplast genomes. The analysis proceeded in three main steps: First, all protein-coding sequences (CDSs) were extracted based on genome annotations. Next, the RSCU value for each synonymous codon was calculated using the formula: RSCU = (observed frequency of a codon)/(expected frequency under equal usage of all synonymous codons for that amino acid). An RSCU value of 1.0 indicates no bias, values >1.0 suggest a codon is preferentially used, and values <1.0 denote underrepresentation (Wang et al., 2018). To facilitate cross-species comparison, heatmaps were constructed using the pheatmap package in R, allowing for a clear visualization of codon usage patterns among different species. This approach eliminates the influence of amino acid composition by standardizing codon counts, thereby accurately reflecting genome-wide codon usage preferences (Perrière and Thioulouse, 2002). The RSCU-based analysis offers insights into the evolutionary pressures acting on chloroplast genomes and helps reveal lineage-specific codon usage strategies. These findings are essential for understanding the molecular evolution, gene expression regulation, and functional optimization of chloroplast-encoded proteins in Rutaceae.

### 2.5 ENC analysis

To assess codon usage diversity, the Effective Number of Codons (ENC) was calculated using the ENC-GC3s analytical approach (Wright, 1990). The ENC value was derived from the formula: ENC = 2 + 9/F_2_ + 1/F_3_ + 5/F_4_ + 3/F_6_, where F_2_ to F_6_ represent the homozygosity (codon usage bias) indices for amino acids encoded by 2 to 6 synonymous codons, respectively. ENC values theoretically range from 20 (indicating extreme codon usage bias) to 61 (indicating no bias). In this study, custom Python scripts were employed to compute the ENC values for each protein-coding gene, along with the GC content at the third codon position (GC3s). Subsequently, ENC-GC3s scatter plots were generated using the R programming environment. These plots incorporated a standard expected curve representing the theoretical relationship between ENC and GC3s under the assumption that codon usage is solely dictated by GC compositional constraints. The extent to which observed data points deviated from this expected curve was used to infer the relative influence of mutational pressure and translational selection on codon usage bias. Genes aligning closely with the theoretical curve were interpreted as being mainly influenced by mutational bias, whereas genes deviating significantly from the curve were considered to be under selection-driven codon usage patterns. This analytical framework enabled a nuanced understanding of the evolutionary forces shaping codon usage in chloroplast genomes.

### 2.6 Neutrality plot analysis

Neutrality plot analysis was conducted to explore the selective forces influencing chloroplast genome evolution in Rutaceae species. For each gene, the average GC content at the first and second codon positions (GC12) and at the third codon position (GC3) were calculated. A GC3-GC12 neutrality plot was then constructed, with each data point representing a gene’s GC content characteristics. Linear regression analysis was applied to assess the correlation between GC12 and GC3. The regression slope was interpreted as follows: a slope close to 1 indicates that mutational pressure predominantly influences the gene’s GC content, while a slope near 0 suggests that natural selection primarily drives the gene’s GC content variation (Tang et al., 2021; Yang et al., 2024). This analysis allowed for the assessment of the relative contributions of mutational bias and selective forces to codon usage in the chloroplast genomes of Rutaceae species.

### 2.7 PR2 plot analysis

PR2 (Parity Rule 2) analysis was employed to examine the evolutionary driving forces behind codon usage preferences in the chloroplast genomes of Rutaceae species. According to the principle of base-pairing equilibrium, under the absence of selection pressures, the third codon position should exhibit an equal distribution of A = T and G = C (Yang et al., 2023a). However, natural selection typically results in a deviation from this equilibrium. To investigate this, the ratios of A3/(A3+T3) and G3/(G3+C3) were calculated for each gene. A two-dimensional scatter plot was constructed, with G3/(G3+C3) on the x-axis and A3/(A3+T3) on the y-axis. The central point (0.5, 0.5) represents the theoretical balance, and the distance of data points from this central point reflects the extent of base composition bias. The plot was divided into four quadrants using the 0.5 reference line, with regions of deviation indicating the presence of natural selection pressures. The analysis, visualized using the ggplot2 package, highlighted distinct selection patterns across species and provided a detailed comparison of codon usage biases across different Rutaceae lineages.

### 2.8 Correspondence analysis

Correspondence analysis (COA) was performed to systematically explore the codon usage patterns in Rutaceae species. Based on the RSCU matrix (excluding amino acids encoded by single codons, such as methionine and tryptophan), each protein-coding sequence was converted into a 59-dimensional vector. Principal component analysis (PCA) was conducted using the FactoMineR package to reduce the dimensionality, and the first two principal components, which accounted for the highest proportion of variation, were extracted. Visualization of the results was achieved through a two-dimensional plot generated by the factoextra package. This analysis revealed the major trends in codon usage variation across species and provided insights into the functional constraints and evolutionary pressures shaping codon usage preferences in the Rutaceae family.

### 2.9 Collinearity analysis

Collinearity analysis was performed across the chloroplast genomes of 28 species. The analysis was visualized using the genoPlotR package in R (v4.1.0). The workflow included the following steps: (1) gene feature information was extracted from the GFF3 annotation files and standardized; (2) genes were categorized based on functional groups (e.g., photosystem, ATP synthase, transcription and translation-related genes), with differential coloring applied to each category; (3) pairwise sequence similarity was calculated using BLASTN; (4) a phylogenetic tree based on the maximum likelihood method (Newick format) was integrated. The results clearly illustrated the conserved quadripartite structure of the chloroplast genome, with gray connecting lines indicating regions of high sequence similarity. This analysis provided critical evidence of genome structure variation, such as rearrangements and inversions, particularly near the inverted repeat (IR) region boundaries, which are linked to phylogenetic differentiation.

### 2.10 Selection pressure analysis

Selection pressure analysis was performed using the Ka/Ks Calculator v2.0 (Wang et al., 2010) to calculate the non-synonymous substitution rate (Ka) and synonymous substitution rate (Ks) for homologous genes, and the Ka/Ks ratio (ω) was subsequently calculated. The ω ratio is used to assess the type of selection: ω > 1 indicates positive selection, reflecting the fixation of adaptive mutations; ω ≈ 1 indicates neutral evolution; and ω < 1 suggests purifying selection, which eliminates deleterious mutations. This analysis provided molecular evidence for understanding the adaptive evolutionary processes of chloroplast-encoded genes in Rutaceae species.

### 2.11 Phylogenetic reconstruction

Phylogenetic analysis was performed using conserved protein-coding genes from the chloroplast genomes. Multiple sequence alignment was carried out with MAFFT v7.450 using the L-INS-i algorithm for accurate alignment of highly variable regions (Katoh and Standley, 2013), and the resulting alignment was concatenated using PhyloSuite v1.2.2 (Zhang et al., 2020). To optimize the alignment quality, trimAl v1.4 (automated1 parameter) was applied to remove poorly aligned positions and divergent regions. The optimal partitioning scheme and substitution models were determined using PartitionFinder v2.1.1 with the Bayesian Information Criterion (BIC) for model selection (Lanfear et al., 2017). Phylogenetic reconstruction was performed using the maximum likelihood method (RAxML-NG v1.2.2) with 1000 bootstrap replicates (Kozlov et al., 2019), and the final phylogenetic tree robustly resolved the evolutionary relationships among Rutaceae species. All analyses were conducted with default parameters unless otherwise specified, and complete parameter settings are detailed in the supplementary methods.

## 3 Results

### 3.1 Chloroplast genome features

**Figure 1:**
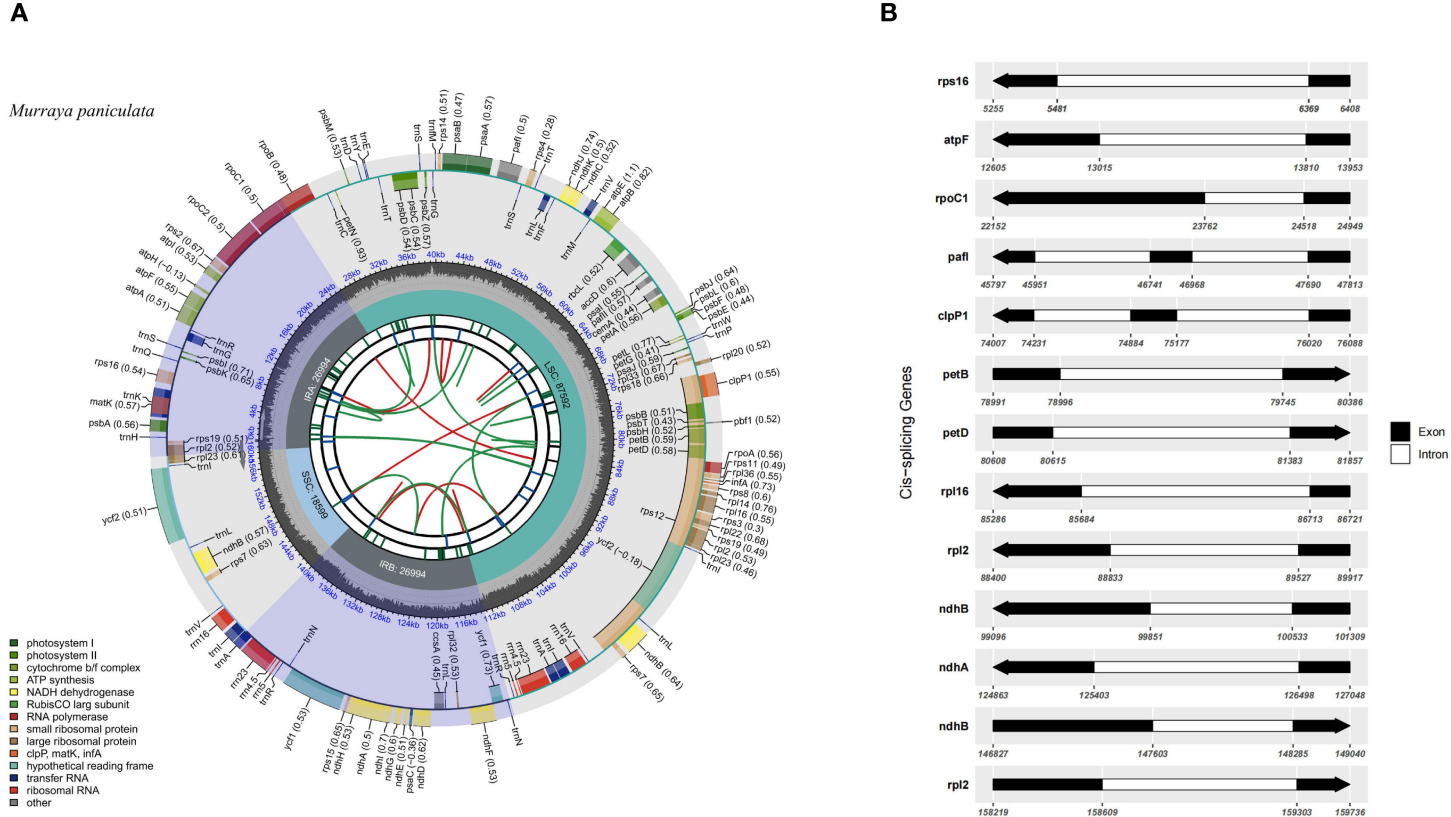
Chloroplast genome features of *M. paniculata.* **(A)** Circular map of the chloroplast genome of *Murraya paniculata*. The map contains six concentric tracks. From center outward: the first track shows dispersed repeats consisting of direct (D, red arcs) and palindromic (P, green arcs) repeats; the second track displays long tandem repeats (short blue bars); the third track shows short tandem repeats or microsatellite sequences (short bars in different colors); the fourth track indicates the small single-copy (SSC), inverted repeat (IRa and IRb), and large single-copy (LSC) regions; the fifth track plots GC content along the genome; the sixth track shows gene distribution. Genes are color-coded by functional classification. Transcription directions for inner and outer genes are clockwise and anticlockwise, respectively. Numbers in parentheses after gene names indicate codon usage bias values. **(B)** Schematic representation of cis-splicing genes in the chloroplast genome. Genes are arranged from top to bottom based on their order in the chloroplast genome. Gene names are shown on the left, and gene structures are displayed on the right. Exons are shown in black, and introns in white. Arrows indicate the transcription direction of genes. Note that the lengths of exons and introns are not drawn to scale.

The complete chloroplast genome of *M. paniculata* exhibits a typical quadripartite structure commonly found in angiosperms, with a total length of 160,179 bp. It consists of a large single-copy (LSC) region of 87,592 bp, a small single-copy (SSC) region of 18,599 bp, and a pair of inverted repeats (IRs), each 26,994 bp in length (**Figure 1A**). The overall GC content of the genome is 38.64%, and the total length of coding regions is 79,755 bp. A total of 131 genes were identified, comprising 86 protein-coding genes, 8 rRNA genes, and 37 tRNA genes. Genes associated with photosynthetic functions—such as *psaA/B*, *psbC/D/E/F*, among others—are predominantly located in the LSC region (**Figure 1A**; **Table 1** and **Supplementary Table S1**). The chloroplast genome map clearly illustrates the spatial distribution and orientation of genes across the four structural regions, as well as the exon–intron architecture of key chloroplast genes. Notably, some genes such as *rps16* contain relatively long intronic regions, which may be involved in transcriptional regulation (**Figures 1A, B**). Comparative analysis of 35 Rutaceae species, including *M. paniculata*, revealed that their chloroplast genome sizes ranged from 155 kb to 161 kb, with GC content varying between 38.17% and 38.83%, and gene counts ranging from 122 to 144. These findings indicate an overall high level of genomic conservation within the family (**Table 1**). Analysis of nucleotide composition showed a consistent AT bias across all species, with average base proportions of A (30.46%), T (31.10%), C (19.58%), and G (18.86%). GC content exhibited a positional gradient among codon sites, following the trend GC1 (mean 46.16%) > GC2 (38.34%) > GC3 (31.83%), with the GC content at third codon positions (GC3s) averaging 28.98%, indicating a pronounced AT preference at these positions (**Supplementary Table S1**). The average length of the coding regions across the 35 chloroplast genomes was 78,245 bp, accounting for 49.38% of the total genome length. In contrast, non-coding regions averaged 68,392 bp. The GC content of coding regions (mean 38.78%) was notably higher than that of non-coding regions (mean 35.08%), suggesting greater structural stability and functional constraint in coding sequences (**Supplementary Table S1**).

**Table 1:** Summary of chloroplast genome features in rutaceae species.

no.	Species	Acc. No	Length	GC%	Protein	rRNA	tRNA	Total gene
1	*Clausena excavata*	KU949003	161172	38.28	86	8	33	127
2	*Glycosmis mauritiana*	KU949004	160131	38.49	85	8	29	122
3	*Glycosmis pentaphylla*	KU949005	159844	38.39	85	8	29	122
4	*Micromelum minutum*	KU949007	160416	38.54	86	8	29	123
5	*Phellodendron amurense*	KY707335	158442	38.38	88	8	37	133
6	*Clausena anisata*	LC794893	159569	38.4	91	8	44	143
7	*Glycosmis citrifolia*	LC794899	159008	38.52	89	8	45	142
8	*Murraya caloxylon*	LC794902	160020	38.46	91	8	45	144
9	*Murraya koenigii*	LC794904	159337	38.58	91	8	43	142
10	*Ruta graveolens*	MN326012	157434	38.83	87	8	37	132
11	*Zanthoxylum nitidum*	MN508801	157253	38.5	87	8	37	132
12	*Zanthoxylum motuoense*	MT990981	158509	38.52	86	8	37	131
13	*Citrus reticulata*	MW147176	160699	38.42	87	8	37	132
14	*Melicope lucida*	MW221969	160407	38.57	86	8	35	129
15	*Zanthoxylum asiaticum*	MW478801	158394	38.47	87	8	37	132
16	*Phellodendron chinense*	MW478802	158490	38.36	87	8	37	132
17	*Tetradium ruticarpum*	MW478803	158762	38.33	87	8	37	132
18	*Tetradium daniellii*	MZ145060	158446	38.33	86	8	37	131
19	*Dictamnus albus*	MZ750957	157139	38.49	87	8	37	132
20	*Murraya paniculata*	This study	160179	38.64	86	8	37	131
21	*Boronia ternata*	OL591162	157247	38.17	87	8	37	132
22	*Brombya platynema*	OL591163	158837	38.29	87	8	37	132
23	*Crowea saligna*	OL591172	155807	38.48	87	8	37	132
24	*Cyanothamnus anemonifolius*	OL591173	155860	38.28	85	8	36	129
25	*Drummondita fulva*	OL591177	157286	38.28	86	8	37	131
26	*Eriostemon australasius*	OL591179	157114	38.39	87	8	37	132
27	*Euodia pubifolia*	OL591181	159341	38.22	87	8	37	132
28	*Halfordia kendack*	OL591187	158159	38.27	87	8	38	133
29	*Leionema ellipticum*	OL591190	157187	38.42	86	8	37	131
30	*Corynonema pinoides*	OL591215	156595	38.49	87	8	37	132
31	*Corynonema pungens*	OL591216	155376	38.46	87	8	37	132
32	*Chorilaena anceps*	OL591221	155363	38.41	82	8	37	127
33	*Clausena lansium*	OL944012	159787	38.67	87	6	37	130
34	*Citrus sinensis*	ON641345	160121	38.48	87	8	37	132
35	*Zanthoxylum avicennae*	OP580971	158506	38.45	87	8	37	132

### 3.2 Simple sequence repeat analysis

**Figure 2:**
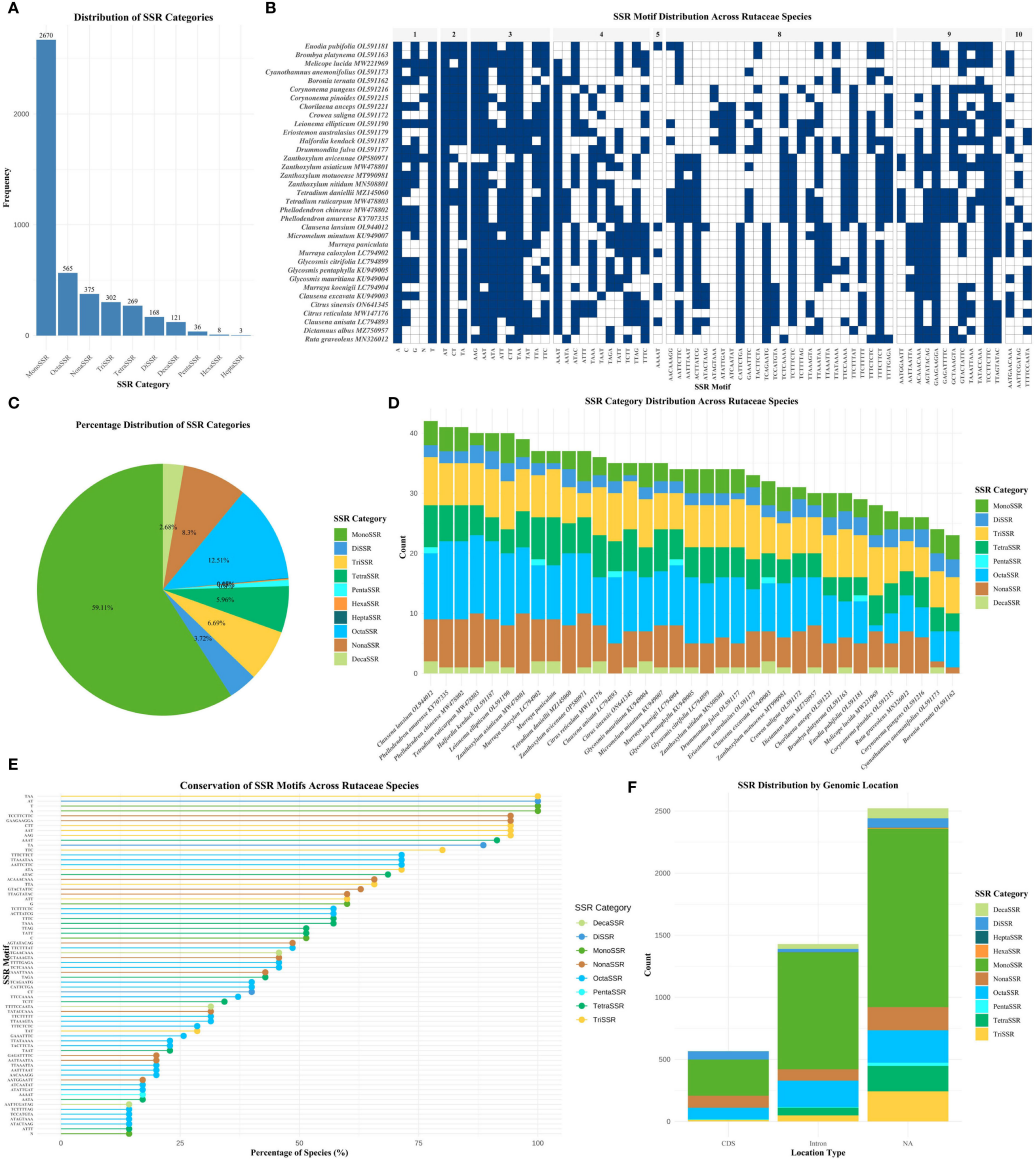
Comprehensive analysis of SSRs in Rutaceae chloroplast genomes. **(A)** Bar chart showing the distribution of different SSR categories based on repeat unit length. **(B)** SSR motif distribution heatmap showing presence/absence patterns across 35 Rutaceae species, with blue indicating presence and white indicating absence, grouped by repeat unit length. **(C)** Pie chart showing the relative distribution of different SSR categories. **(D)** Stacked bar chart displaying SSR category distribution across different Rutaceae species. **(E)** Scatter plot illustrating SSR motif conservation levels across species, with bubble size indicating species count and color representing SSR category. **(F)** Stacked bar chart showing SSR distribution across different genomic locations. SSR categories are defined by repeat unit length: MonoSSR (1 bp), DiSSR (2 bp), TriSSR (3 bp), TetraSSR (4 bp), PentaSSR (5 bp), HexaSSR (6 bp), HeptaSSR (7 bp), OctaSSR (8 bp), NonaSSR (9 bp), DecaSSR (10 bp), and ExtendedSSR (>10 bp).

The analysis of SSRs across the chloroplast genomes of 35 Rutaceae species revealed distinct patterns of distribution and evolutionary significance. A total of 4,517 SSR loci were identified, with their abundance and composition exhibiting substantial variation among species (**Figures 2A, B**; **Supplementary Table S2**). Mononucleotide repeats (MonoSSRs) were the most prevalent type, accounting for 59.11% of all SSRs, followed by octanucleotide (OctaSSR, 12.51%) and nonanucleotide repeats (NonaSSR, 8.30%). In contrast, hexanucleotide (HexaSSR, 0.18%) and heptanucleotide repeats (HeptaSSR, 0.07%) were exceedingly rare, together comprising less than 1% of total SSRs (**Figure 2C**). Species-specific differences in SSR composition were observed. For instance, *Leionema ellipticum* exhibited a markedly higher proportion of MonoSSRs compared to *Eriostemon australasius* (**Figure 2D**). The majority of SSR motifs displayed a pronounced AT richness, while GC-rich repeats—such as those composed of C or G nucleotides—were relatively uncommon. This strong AT bias aligns with the overall base composition of Rutaceae chloroplast genomes, which exhibit average AT contents ranging from 61.17% to 61.83%, and may reflect underlying mutational biases or selective pressures (**Figure 2E**). The genomic distribution of SSRs was non-random, with the majority localized to non-coding regions. Specifically, 55.83% of SSRs were found in intergenic spacer (IGS) regions, 31.61% within introns, and only 12.55% in coding sequences (CDS) (**Figure 2F**). This pattern suggests that SSRs are more likely to accumulate in genomic regions subject to weaker selective constraints. The low SSR density in coding regions may result from purifying selection acting to prevent frameshift mutations or disruptions of protein function.

### 3.3 Relative synonymous codon usage analysis

**Figure 3:**
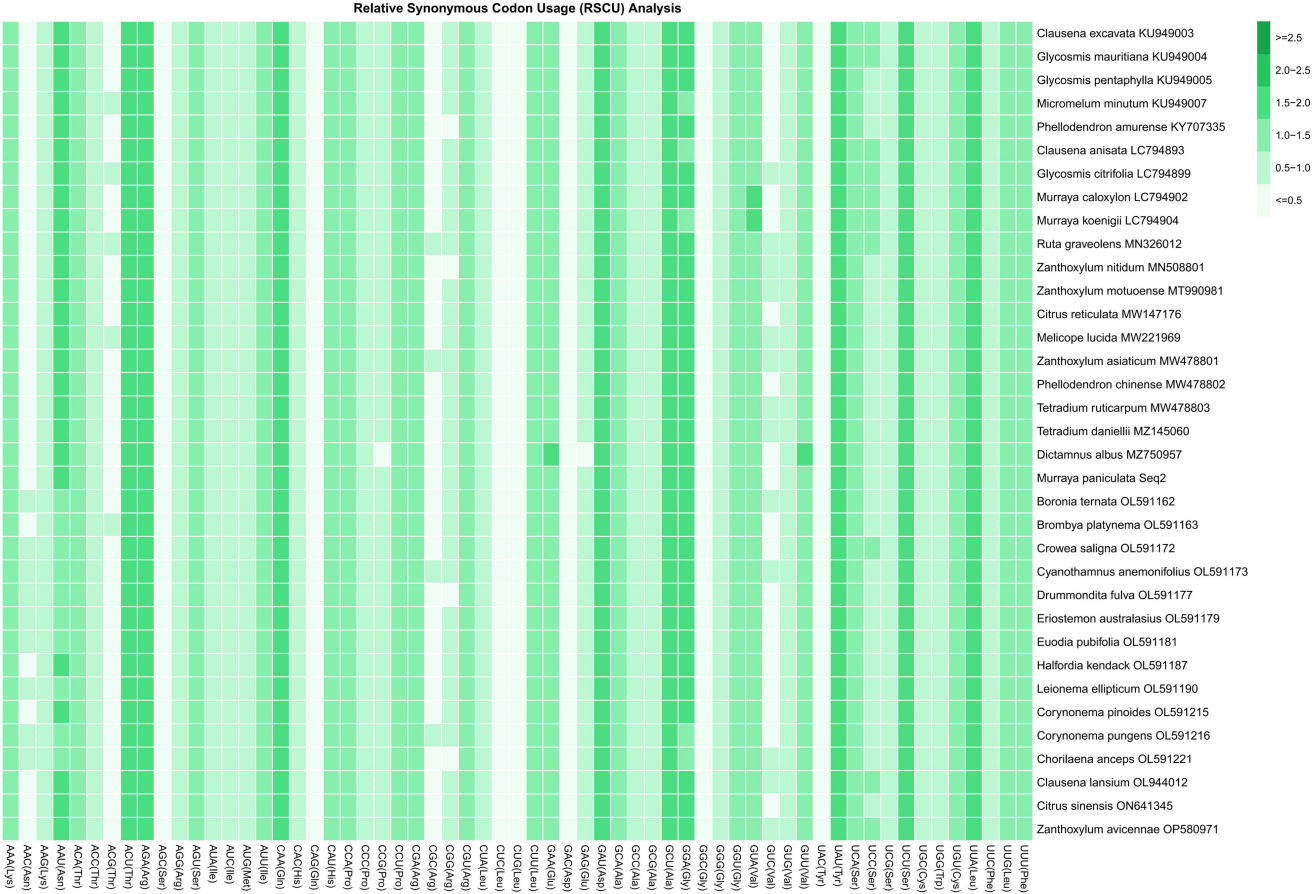
Relative synonymous codon usage (RSCU) values for all codons across 35 Rutaceae species. Each species is labeled with its scientific name and corresponding GenBank accession number. Codons are presented alongside their associated amino acids [Codon (Amino Acid)]. RSCU values represent the relative frequency of codon usage: values >1 indicate codons used more frequently than expected under uniform synonymous usage; values <1 indicate less frequent usage; values =1 indicate codons used at expected frequency. This analysis provides a comprehensive overview of codon usage patterns in Rutaceae, offering insights into underlying evolutionary pressures and preferences for translational efficiency.

The RSCU analysis revealed notable codon usage bias across the chloroplast genomes of 35 Rutaceae species. The RSCU values ranged from 0.386 to 1.797 (**Figure 3**; **Supplementary Table S3**), confirming that all investigated species exhibit non-random usage of synonymous codons. Among the 20 amino acids, tryptophan (Trp, encoded solely by UGG) and methionine (Met, encoded solely by AUG) are represented by single codons, while the remaining 18 amino acids are encoded by two to six synonymous codons. Three codons showed strong preferential usage with RSCU values greater than 1.6: AGA (Arg, RSCU = 1.753), UUA (Leu, RSCU = 1.732), and GCU (Ala, RSCU = 1.702). Conversely, 19 codons were significantly underrepresented (RSCU < 0.6), including UAC (Tyr, RSCU = 0.406), AGC (Ser, RSCU = 0.417), and GGC (Gly, RSCU = 0.424). An additional 13 codons exhibited neutral usage patterns, with RSCU values between 0.8 and 1.2. Comparative analysis among species indicated that closely related genera (e.g., *Citrus* and *Murraya*) displayed highly similar RSCU profiles, suggesting conserved codon usage preferences within these lineages. In contrast, more distantly related taxa such as *Boronia* and *Zanthoxylum* exhibited divergent codon usage patterns, highlighting lineage-specific evolutionary trajectories. These findings demonstrate that codon usage bias in Rutaceae is both conserved within genera and variable across the family, reflecting the combined effects of mutational pressure and selection.

### 3.4 Effective number of codons plot analysis

**Figure 4:**
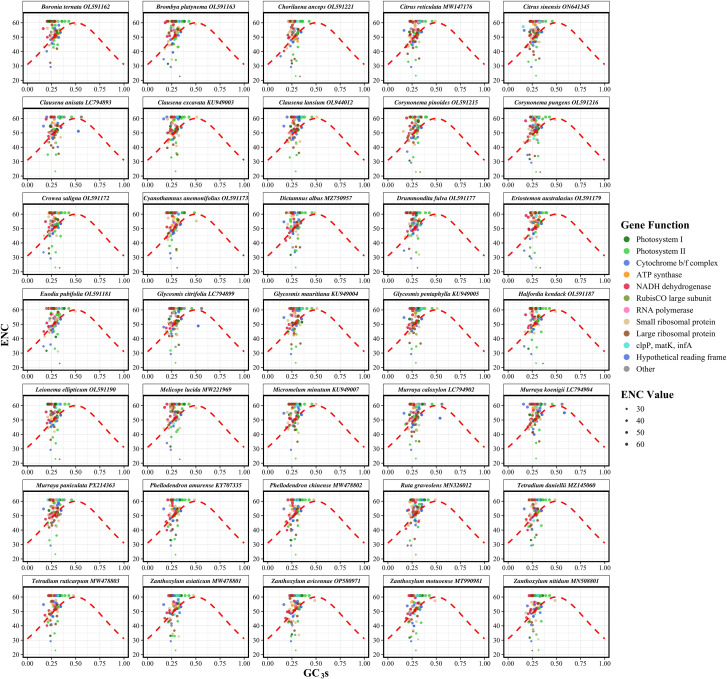
Effective number of codons (ENC) plotted against GC content at the third codon position (GC3s) for chloroplast protein-coding genes in 35 Rutaceae species. Each point represents a single gene, with point size proportional to its ENC value. The red dashed line indicates the theoretical expectation under neutral evolution, where codon usage bias is solely dictated by GC3s. Genes falling below the curve exhibit stronger codon usage bias than expected from compositional constraints alone, implying the action of natural selection. Species are displayed in faceted panels arranged alphabetically to facilitate cross-species comparison within Rutaceae. Deviations from the expected curve reflect the influence of non-neutral evolutionary forces shaping codon usage patterns.

The ENC analysis revealed significant variation in codon usage bias across the chloroplast genomes of Rutaceae species. The ENC values among species ranged from 51.00 to 53.78, with an average of 52.10, while the GC content at the third codon position (GC3s) varied from 0.275 to 0.294, averaging 0.281 (**Supplementary Table S4**). These values suggest that, although codon usage in Rutaceae chloroplast genomes is not highly constrained, a moderate degree of bias exists. The ENC-GC3s scatter plot showed that the vast majority of gene data points were positioned above the expected theoretical curve (**Figure 4**), which represents codon usage governed solely by GC3s composition under neutral evolutionary conditions. The average ENC value across all species was 52.10, which is markedly lower than the neutrality expectation of 61.0 (**Supplementary Table S4**). This deviation, observed consistently across the 35 Rutaceae species, supports the conclusion that codon usage in chloroplast protein-coding genes is not solely dictated by mutational bias but is also influenced by natural selection. This consistent deviation across species indicates that codon usage in chloroplast protein-coding genes is not solely dictated by mutational bias but is also influenced by natural selection and other evolutionary forces. Species within the genera *Citrus* (e.g., *C. reticulata* and *C. sinensis*) and *Murraya* exhibited highly similar ENC-GC3s distribution patterns, reflecting conserved codon usage traits within these lineages. In contrast, more distantly related genera such as *Boronia ternata* and *Zanthoxylum nitidum* displayed distinct patterns, suggesting that codon usage bias has also undergone lineage-specific adaptive divergence.

### 3.5 Neutrality plot analysis

**Figure 5:**
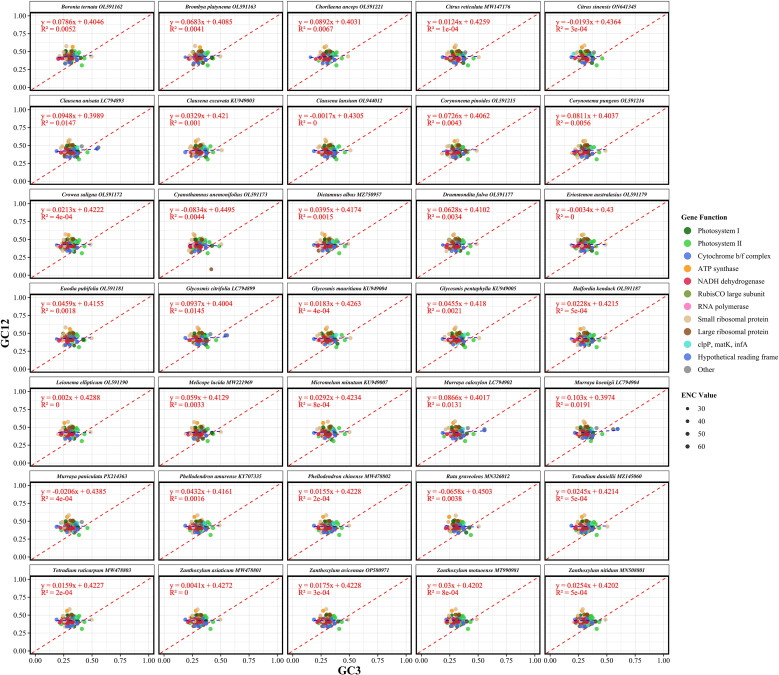
Neutrality analysis of codon usage in chloroplast protein-coding genes from 35 Rutaceae species. Each panel illustrates the relationship between GC content at the third codon position (GC3) and the mean GC content at the first and second codon positions (GC12). Each point corresponds to a single gene, with point size proportional to its effective number of codons (ENC). The blue dashed line represents the fitted regression line for each species, while the red dashed line denotes the neutral expectation (GC12 = GC3). Deviations from the red line suggest non-neutral evolutionary processes, where a strong positive correlation between GC3 and GC12 implies mutational bias as the dominant force, rather than selection on codon usage. Regression equations and R² values are shown in each panel to indicate the strength of the GC3–GC12 relationship. Species are arranged alphabetically in faceted panels to enable comparative assessment across the Rutaceae family.

To investigate the evolutionary forces shaping codon usage in Rutaceae chloroplast genomes, neutrality plot analysis was performed based on nucleotide composition at different codon positions. Among the 3,041 protein-coding genes analyzed, GC content at the third codon position (GC3) ranged from 0.3054 to 0.3238 (mean = 0.3110), whereas the average GC content at the first and second codon positions (GC12) ranged from 0.4294 to 0.4308 (mean = 0.4295). This positional gradient indicates that codon sites are subject to heterogeneous evolutionary constraints. Linear regression analysis between GC3 and GC12 showed slopes ranging from –0.0834 to 0.1030 (mean = 0.0326), with relatively low R² values (0 to 0.0191, mean = 0.0033) (**Figure 5**; **Supplementary Table S5**). These results suggest that natural selection, rather than mutational bias, plays a dominant role in shaping codon usage patterns in the chloroplast genomes of Rutaceae species.

### 3.6 PR2 plot analysis

**Figure 6:**
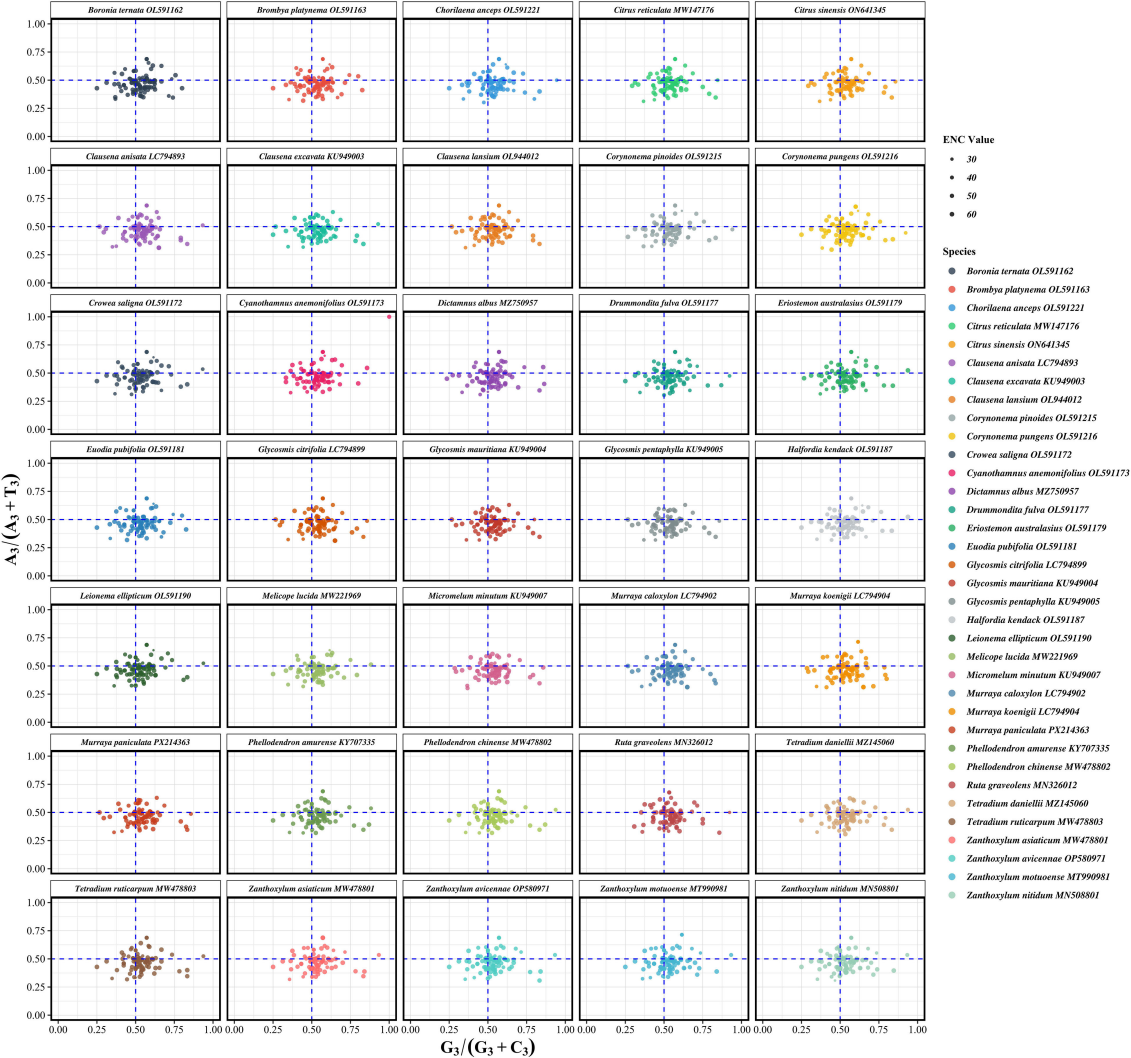
Parity rule 2 (PR2) analysis of chloroplast protein-coding genes in 35 Rutaceae species. Each panel depicts the relationship between the G3/(G3+C3) and A3/(A3+T3) ratios for individual genes within each species. Each point represents a single gene, with point size proportional to its effective number of codons (ENC). The blue dashed lines represent the theoretical expectation under PR2 (0.5 for both axes), which assumes equal usage of complementary nucleotides (A = T, G = C) at the third codon position under neutral conditions. Deviations from the center point (0.5, 0.5) indicate violations of PR2 symmetry and suggest the influence of factors such as transcriptional or replicational strand bias, or selective constraints. Species are arranged alphabetically in faceted panels to support comparative analysis across the Rutaceae family.

PR2 (Parity Rule 2) analysis was conducted to assess potential asymmetry in base usage at the third codon position, thereby evaluating the relative impact of mutation versus selection on codon usage. Under neutral expectations, the frequencies of complementary bases (A = T and G = C) should be balanced. Deviations from this symmetry suggest the influence of selective constraints (Parvathy et al., 2022). For the 35 Rutaceae species analyzed, the average ratio of A3/(A3 + T3) was 0.4694 ± 0.0747, significantly lower than the neutral expectation of 0.5 (p < 0.01), indicating a general preference for T over A. Similarly, the average G3/(G3 + C3) ratio was 0.5369 ± 0.0993, showing a moderate bias toward G over C (**Figure 6**; **Supplementary Table S6**). In PR2 plots, most gene data points clustered in the fourth quadrant (A3/(A3 + T3) < 0.5 and G3/(G3 + C3) > 0.5), supporting the presence of directional base usage bias. This asymmetric pattern was highly conserved across phylogenetic branches within Rutaceae, implying a shared selective landscape. At the genus level, codon usage asymmetry also exhibited lineage-specific characteristics. For example, *Citrus* species (n = 174) showed G3/(G3 + C3) = 0.5375 and A3/(A3 + T3) = 0.4708, while *Murraya* species (n = 268) exhibited G3/(G3 + C3) = 0.5406 and A3/(A3 + T3) = 0.4672. These subtle differences suggest genus-specific patterns of codon bias potentially shaped by ecological or functional constraints.

### 3.7 COA analysis

**Figure 7:**
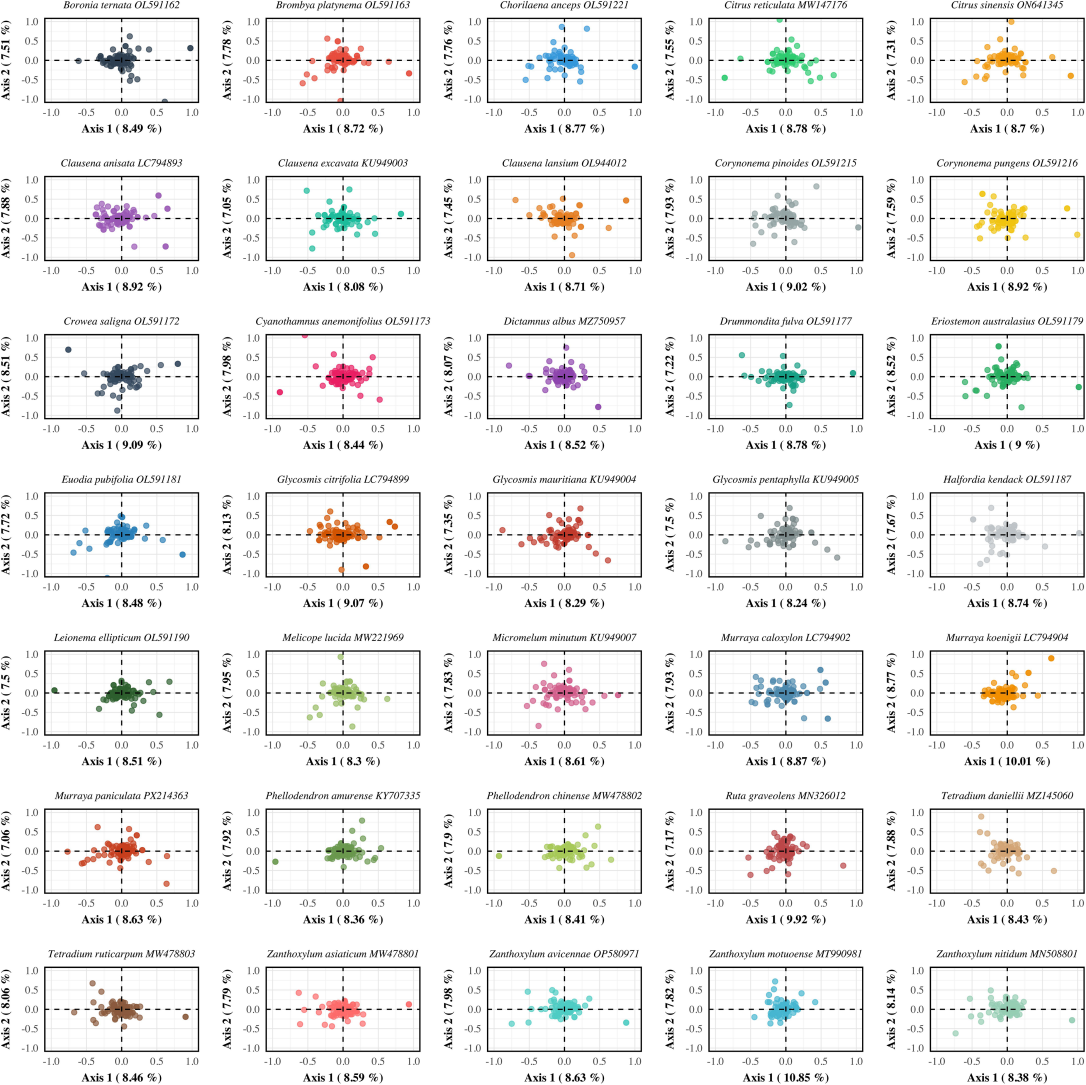
Correspondence analysis (COA) of codon usage bias in chloroplast protein-coding genes across 35 Rutaceae species. Each panel represents a single species and displays the distribution of genes in a two-dimensional COA space. Each point corresponds to a gene, positioned based on its codon usage pattern as determined by relative synonymous codon usage (RSCU) values. The first two axes represent the major sources of variation in codon usage among genes, with the percentages along Axis 1 and Axis 2 indicating the proportion of total variance explained by each dimension. This analysis reveals species-specific codon usage patterns and helps identify the primary factors shaping synonymous codon selection in Rutaceae chloroplast genomes. Species are arranged alphabetically to facilitate systematic comparison across different lineages within the family.

To explore the primary trends and potential driving factors of synonymous codon usage, Correspondence Analysis (COA) was performed on the chloroplast genomes of 35 Rutaceae species. The first and second axes together accounted for 16.54% of the total variation in codon usage bias (CUB), with axis 1 explaining 8.76% and axis 2 explaining 7.78% (**Figure 7**; **Supplementary Table S7**). These results indicate that codon usage in Rutaceae chloroplast genomes is shaped by multiple interacting factors, including mutational bias, translational selection, and possibly functional constraints. Although interspecific differences in codon usage patterns were evident, most species exhibited similar distribution trends. For example, most coding sequences (CDSs) from *Murraya* and *Citrus* clustered near the center of the plot, reflecting a relatively conserved CUB landscape within these genera. In contrast, a small subset of genes appeared at the periphery of the plot, indicating distinct codon usage preferences potentially linked to differential gene expression or lineage-specific selection.

### 3.8 Collinearity analysis

**Figure 8:**
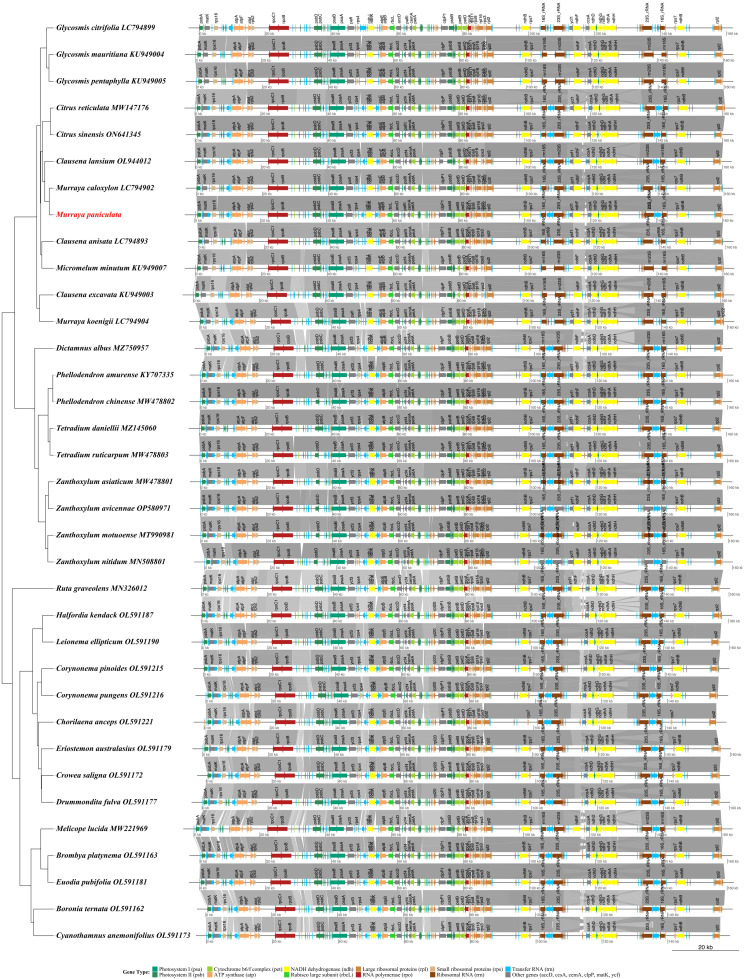
Synteny analysis of chloroplast genomes from 35 Rutaceae species. The phylogenetic tree inferred from complete chloroplast genome sequences is shown on the left, while linear representations of genome structures are displayed on the right. Gene types are color-coded as follows: green shades for photosystem genes (*psa*, *psb*), yellow-green for cytochrome b_6_/f complex genes (*pet*), orange for ATP synthase genes (*atp*), yellow for NADH dehydrogenase genes (*ndh*), light green for the Rubisco large subunit gene (*rbcL*), brown shades for ribosomal protein genes (*rpl*, *rps*), red for RNA polymerase genes (*rpo*), blue for transfer RNA genes (*trn*), dark brown for ribosomal RNA genes (*rrn*), and gray for other functional genes. Gray connecting lines represent conserved syntenic blocks between genomes. The scale bar indicates genome length in kilobases (kb).

A comprehensive collinearity analysis of chloroplast genomes from 35 Rutaceae species was conducted to systematically reveal conserved structural features and evolutionary relationships within the family. The results showed that Rutaceae chloroplast genomes maintain a highly conserved quadripartite structure (LSC–IRb–SSC–IRa), with gene order largely preserved across species. For instance, the genomic locations of key photosynthetic genes (*psaA*, *psaB*) and ATP synthase-related genes (*atpA*, *atpF*) were completely conserved across all species. Closely related taxa exhibited highly collinear patterns in gene order, orientation, and arrangement, confirming the slow evolutionary rate of chloroplast genome structure among closely related Rutaceae lineages. Phylogenetic reconstruction further clarified intrafamilial relationships within Rutaceae: Subtropical fruit-bearing genera such as *Citrus*, *Murraya*, and *Clausena* clustered into a monophyletic group, while temperate woody genera like *Zanthoxylum*, *Tetradium*, and *Phellodendron* formed distinct clades, and Australian-endemic genera such as *Boronia* and *Crowea* were positioned basally in the phylogenetic tree. The collinearity analysis showed that genomic regions encoding photosynthesis-related genes, ribosomal proteins, and tRNAs were highly conserved, but gene rearrangements were also observed near the IR boundaries in certain species (**Figure 8**). These findings not only confirm the overall structural conservation of Rutaceae chloroplast genomes, but also highlight subtle structural variations that have arisen during long-term evolutionary divergence.

### 3.9 Patterns of selection pressure across chloroplast genes

**Figure 9:**
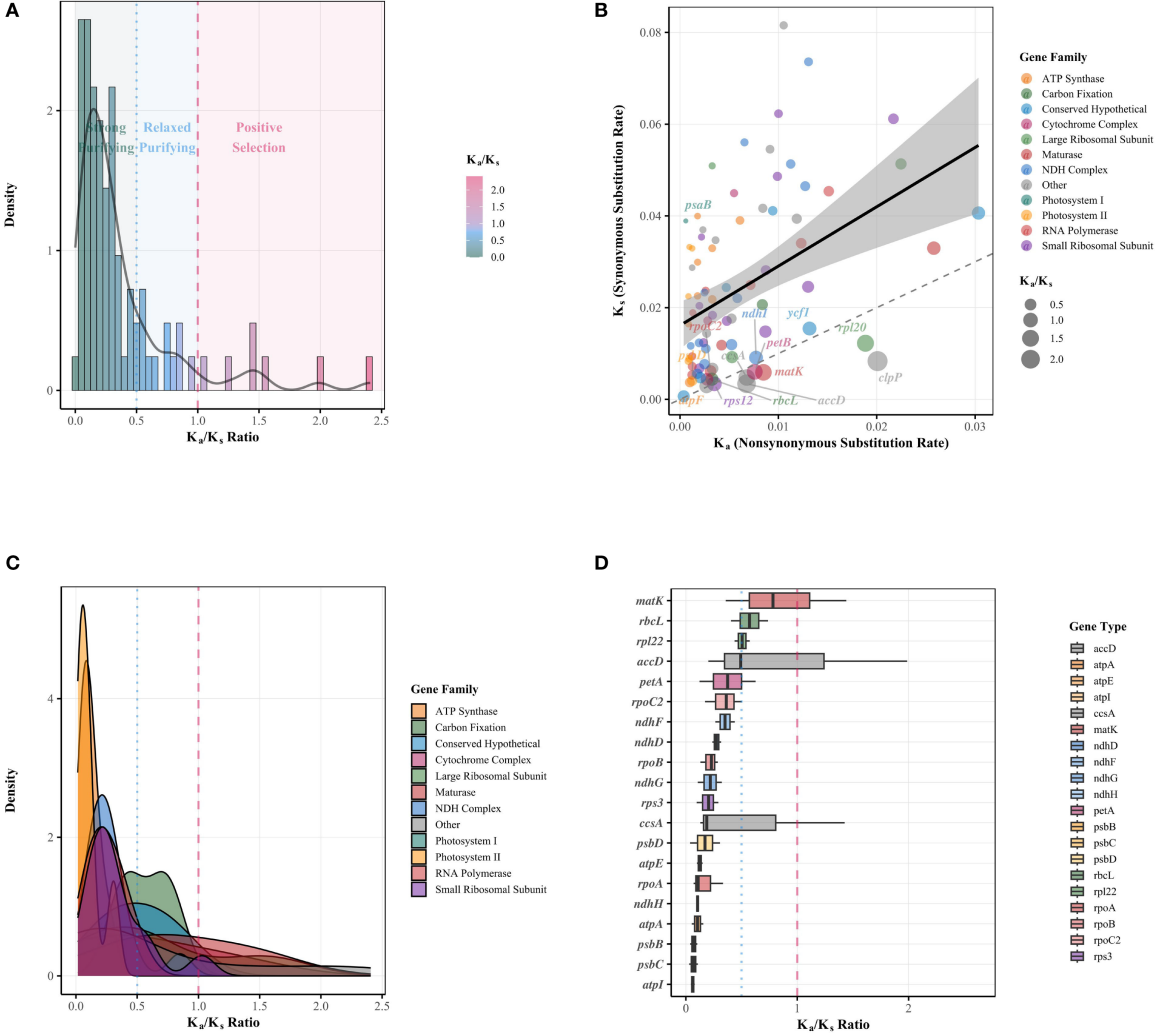
Comprehensive Ka/Ks analysis of chloroplast genes in Rutaceae species. **(A)** Histogram with overlaid density curve depicting the overall distribution of Ka/Ks ratios across all analyzed genes. Background shading indicates regions under different selection regimes: green for strong purifying selection (Ka/Ks ≤ 0.5), blue for relaxed purifying selection (0.5 < Ka/Ks ≤ 1.0), and red for positive selection (Ka/Ks > 1.0). The black curve represents the smoothed density distribution. **(B)** Scatter plot showing the relationship between synonymous (Ks) and nonsynonymous (Ka) substitution rates for individual chloroplast genes. Points are color-coded by functional gene family and scaled by Ka/Ks ratio. The dashed diagonal line represents the neutral evolution threshold (Ka = Ks), while the solid line with shaded area indicates the fitted linear regression and its 95% confidence interval. Italicized gene names highlight representative genes with notable Ka/Ks values from each functional category. **(C)** Density plots showing the distribution of Ka/Ks ratios across different functional gene families. Each colored curve represents a distinct gene family, with overlapping areas revealing differences in evolutionary constraints. Vertical dashed red and dotted blue lines indicate thresholds for neutral evolution and strong purifying selection, respectively. **(D)** Box plots illustrating the distribution of Ka/Ks ratios for individual chloroplast genes, ordered by their median Ka/Ks values. Boxes represent interquartile ranges with medians indicated by horizontal lines. The dashed red line marks the neutral evolution threshold (Ka/Ks = 1), and the dotted blue line marks the threshold for strong purifying selection (Ka/Ks = 0.5). Gene names are italicized.

In this study, a systematic Ka/Ks analysis was performed on protein-coding genes from the chloroplast genomes of 35 Rutaceae species, revealing patterns of evolutionary selection across different functional gene categories (**Figure 9**). Among the 83 gene comparisons analyzed, 77% of genes were found to be under strong purifying selection (Ka/Ks < 0.5), with core photosynthetic genes—such as *psaB* and *psbA/B/C/D*—exhibiting the strongest selective constraints (Ka/Ks < 0.05), whereas genes such as *clpP*, *accD*, and *rpl20* showed significant signals of positive selection (Ka/Ks > 1.5) (**Figure 9A**; **Supplementary Table S8**). Functional comparisons indicated that ribosomal protein genes had a significantly higher average Ka/Ks ratio (0.42) than ATP synthase genes (0.11), reflecting varying levels of evolutionary constraint among functional modules (**Supplementary Table S8**). Scatter plot analysis of Ka/Ks values revealed that most genes were located below the neutral evolution threshold (**Figure 9B**), with photosystem II and ATP synthase genes showing the strongest signatures of purifying selection, whereas NDH complex genes displayed a broader range of Ka/Ks values, with some members approaching the threshold for neutral evolution (**Figure 9C**). Notably, conserved hypothetical proteins and other chloroplast genes exhibited the most diverse selection pressure profiles (**Figure 9C**). Gene-by-gene Ka/Ks distribution analysis further demonstrated substantial heterogeneity in selection pressure across different chloroplast genes (**Figure 9D**). Overall, quantitative analysis showed that 77.1% of the genes were under strong purifying selection (Ka/Ks < 0.5), 14.5% exhibited weak purifying selection (0.5 < Ka/Ks ≤ 1.0), and only 8.4% of gene comparisons showed potential signs of positive selection (Ka/Ks > 1.0) (**Figure 9A**; **Supplementary Table S8**), with most positive selection signals associated with specific members of the NDH complex and hypothetical proteins.

### 3.10 Phylogenetic analysis of *Murraya* species

**Figure 10:**
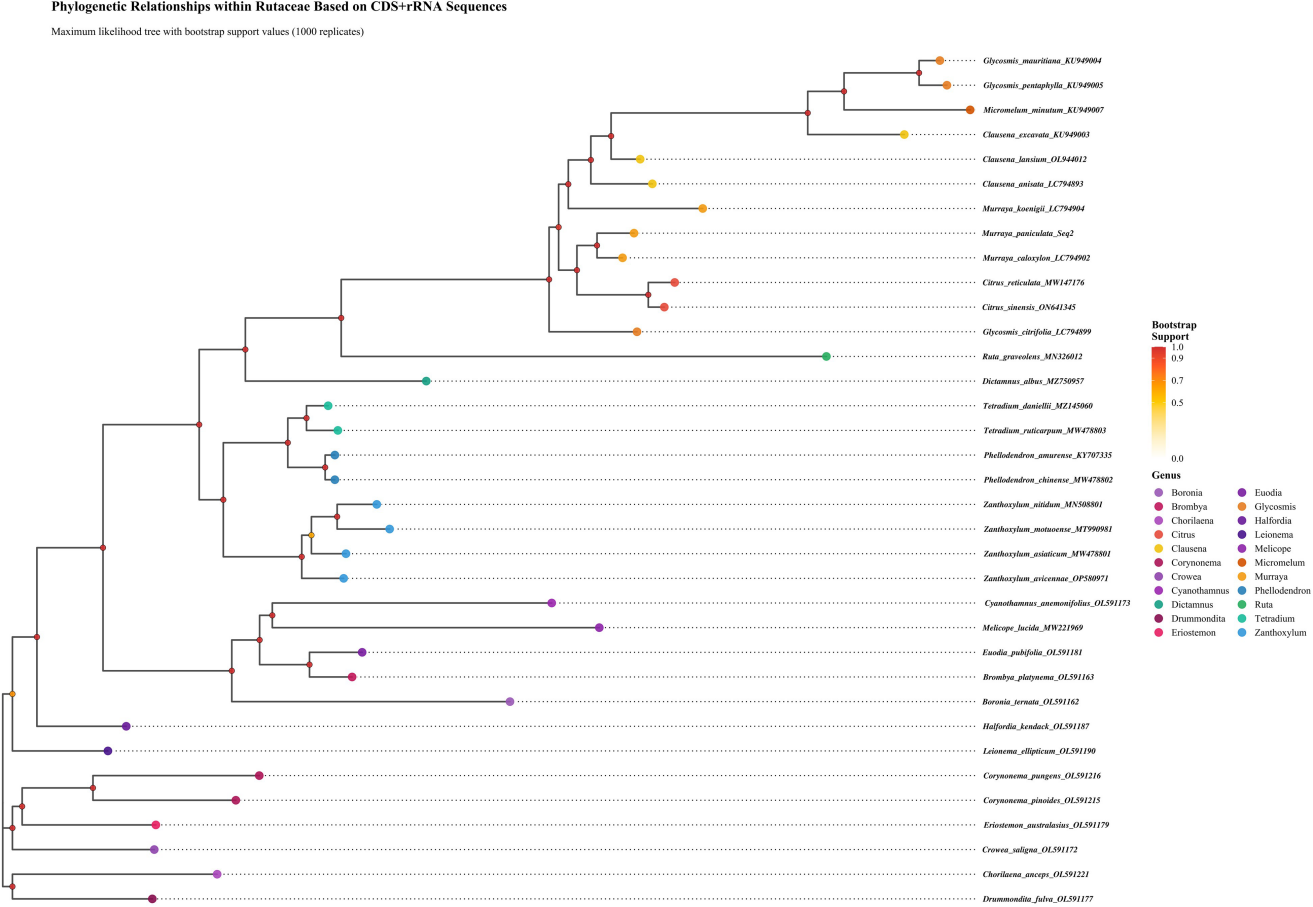
Phylogenetic relationships among Rutaceae species inferred from chloroplast protein sequences. The rectangular phylogenetic tree was constructed from concatenated chloroplast target sequences, with bootstrap support values shown at nodes. Branch lengths correspond to evolutionary distances.

A phylogenetic analysis was conducted on 35 species within the Rutaceae family, based on complete chloroplast genome data. The maximum likelihood phylogenetic tree (**Figure 10**) revealed that all studied species formed well-supported monophyletic clades, validating their current taxonomic placements. The phylogenetic topology further indicated that *Murraya* and *Citrus* species clustered into a highly supported clade, suggesting a close genetic relationship between the two genera. This clade was further grouped with *Clausena*, *Glycosmis*, and *Micromelum* to form a larger evolutionary lineage, reflecting close phylogenetic affinities among these genera. Multiple lines of evidence supported the reliability of the phylogenetic reconstruction: First, most internal nodes were associated with high bootstrap support values; Second, genetic distance analysis among species suggested relatively recent divergence events, with an average Ks value of 0.0241. Collectively, these findings indicate that the studied Rutaceae species may have undergone relatively recent adaptive radiation events. The phylogenetic framework established in this study provides molecular evidence for elucidating the evolutionary history and taxonomic relationships of economically important Rutaceae crops. The results confirm that chloroplast genome data serve as effective molecular markers for phylogenetic inference and species identification within the Rutaceae. These findings have significant theoretical implications for germplasm conservation, cultivar improvement, and the sustainable utilization of Rutaceae genetic resources.

## 4 Discussion

By comparing chloroplast genomes of 35 Rutaceae species, this study systematically elucidated the evolutionary characteristics of the family in terms of genome structure, repeat sequence distribution, and selection pressure. The results confirmed that Rutaceae chloroplast genomes exhibit the typical and highly conserved quadripartite structure (LSC–IRb–SSC–IRa), with genome size (155 – 161 kb), GC content (38.17 – 38.83%), and gene number (122 – 144) all falling within the typical range observed in angiosperm chloroplast genome (Wicke et al., 2011; Jansen and Ruhlman, 2012; Daniell et al., 2016). Notably, variation at the boundaries of the inverted repeat (IR) regions and the strong AT-rich preference in mononucleotide SSRs (accounting for 59.11%) reflect the plasticity of non-coding regions in genome evolution, a pattern consistent with recent findings in *Citrus*, *Glycosmis*, and the Leguminosae family (Sabir et al., 2014; Carbonell-Caballero et al., 2015; Zhao et al., 2022). These observations are further supported by recent studies in other plant families, such as the Zingiberaceae and Gnetales, which also highlight the role of IR boundary shifts and SSR accumulation in driving structural diversity and adaptation (Yang et al., 2023b, 2024). Collinearity analysis further revealed that gene order of key photosynthetic genes (e.g., *psaA/B*, *psbC/D*) and ATP synthase genes (*atpA/F*) was completely conserved across species, whereas lineage-specific rearrangements detected near IR boundaries may be associated with ecological adaptation within the family (Jansen et al., 2007; Mower et al., 2010). These structural features provide important insights into the mechanisms of chloroplast genome stability in Rutaceae and their utility as molecular markers for phylogenetic reconstruction.

Analysis of codon usage bias (CUB) in chloroplast genomes offers new perspectives on the evolutionary forces acting on Rutaceae. We found that codon usage patterns in Rutaceae chloroplast genomes were markedly heterogeneous (RSCU values ranging from 0.386 to 1.797), with highly preferred codons (e.g., AGA, UUA, GCU) ending in A or T, consistent with the overall AT-rich nature of these genomes (mean AT content = 61.56%). These findings support the dominant role of mutational pressure in shaping codon usage bias (Behura and Severson, 2013). However, both ENC-GC3s analysis and neutrality plots (GC12–GC3) showed that empirical data points deviated significantly from the theoretical neutral curve (mean regression slope = 0.0326, R² = 0.0033), indicating that natural selection also plays a significant role in constraining codon usage (Morton, 2003), particularly for highly expressed photosynthesis-related genes such as *psbA* and *rbcL*. These results are consistent with recent findings from chloroplast genomes of Zingiberaceae and Gnetales species, suggesting that chloroplasts may enhance environmental adaptability through translational efficiency optimization (Yang et al., 2023b, 2024). PR2 analysis further revealed asymmetry in third-position base usage (A3/T3 = 0.4694, G3/C3 = 0.5369), a deviation from the neutral expectation (0.5) that remained highly conserved across Rutaceae lineages, possibly reflecting an evolutionary equilibrium shaped by long-term selective pressure (Parvathy et al., 2022). These multidimensional CUB patterns offer molecular evidence for dissecting adaptive evolutionary mechanisms in Rutaceae.

The observed genomic patterns in Rutaceae chloroplast genomes likely reflect adaptations to the family’s remarkably diverse ecological niches and climatic conditions. Rutaceae species exhibit extraordinary ecological diversity, spanning tropical rainforests (e.g., *Murraya* and *Clausena* species in Southeast Asia), Mediterranean climates (e.g., *Citrus* species), temperate deciduous forests (e.g., *Zanthoxylum* and *Tetradium* species), and arid Australian environments (e.g., *Boronia* and *Crowea* species). The pronounced AT-rich bias in SSRs (59.11% mononucleotide repeats) and the consistent deviation from neutral codon usage patterns may represent genomic signatures of adaptation to temperature stress and UV radiation exposure. AT-rich regions in chloroplast genomes have been associated with enhanced thermal stability of DNA-protein interactions and improved photosystem efficiency under high-temperature conditions, which would be particularly advantageous for tropical and subtropical Rutaceae lineages. The genus-specific codon usage patterns we observed may reflect lineage-specific environmental pressures. For instance, the subtropical *Citrus*-*Murraya* clade, which predominantly occupies monsoon-influenced regions with high temperature variability, shows distinct codon preferences compared to temperate *Zanthoxylum* species, which experience pronounced seasonal temperature fluctuations. These differences in synonymous site evolution could facilitate optimization of translational efficiency under different temperature regimes, as has been documented in other plant families subjected to contrasting climatic conditions. Similarly, the Australian-endemic genera (*Boronia*, *Crowea*) occupy some of the most arid and climatically variable environments within Rutaceae’s range, and their basal phylogenetic position combined with distinct codon usage signatures may reflect ancient adaptations to drought stress and extreme temperature fluctuations characteristic of the Australian continent. The positive selection signals detected in genes such as *clpP*, *accD*, and *rpl20* may be particularly relevant to environmental adaptation. The *clpP* gene encodes a protease involved in chloroplast protein quality control, and positive selection on this gene could reflect adaptation to oxidative stress conditions prevalent in high-light, high-temperature environments typical of many Rutaceae habitats. Similarly, *accD* encodes acetyl-CoA carboxylase, a key enzyme in fatty acid biosynthesis that is crucial for maintaining membrane fluidity under temperature stress—a critical adaptation for species spanning tropical to temperate climatic zones. The ribosomal protein gene *rpl20*, which showed signatures of positive selection, could be involved in fine-tuning translational efficiency under varying environmental conditions, particularly important for species experiencing seasonal temperature and light fluctuations.

Selection pressure analysis revealed a complex relationship between gene functional divergence and adaptive evolution in Rutaceae chloroplast genomes. Ka/Ks analysis indicated that 77.1% of genes were under strong purifying selection (Ka/Ks < 0.5), with core photosynthesis genes such as *psbA* and *psaB* showing the strongest selective constraints (Ka/Ks < 0.05), which is consistent with their essential roles in maintaining photosynthetic efficiency (Wang et al., 2010). Notably, genes such as *clpP*, *accD*, and *rpl20* exhibited significant signals of positive selection (Ka/Ks > 1.5), suggesting their involvement in the adaptive divergence of Rutaceae species. This observation aligns with recent findings in Leguminosae and Zingiberaceae, indicating that certain functional modules in the chloroplast genome may be driven by positive selection to facilitate adaptation to diverse ecological niches (Li et al., 2019; Yang et al., 2023b). Selection pressure varied notably among functional categories: ribosomal protein genes (mean Ka/Ks = 0.42) were under weaker constraint than ATP synthase genes (0.11), reflecting a gradient of mutational tolerance across cellular functions (Hurst, 2002). NDH complex genes exhibited the most diverse selection profiles, with some members approaching the threshold of neutral evolution, possibly due to their functional redundancy in cyclic electron transport. These findings offer new insights into the evolutionary dynamics of chloroplast genomes in Rutaceae, particularly by identifying positively selected genes that may serve as targets for future research into adaptive molecular evolution.

The phylogenetic tree constructed in this study provides essential molecular evidence for resolving taxonomic relationships within the Rutaceae. The maximum likelihood tree based on complete chloroplast genome data strongly supports the monophyly of *Murraya* and *Citrus*, consistent with their shared morphological characteristics (e.g., pinnate compound leaves and aromatic flowers) and ecological adaptation to subtropical environments (Carbonell-Caballero et al., 2015). Notably, the clade comprising *Clausena*, *Glycosmis*, and *Micromelum* forms a sister lineage to the *Murraya–Citrus* branch, suggesting that these genera may share key evolutionary innovations. Compared with traditional phylogenetic analyses based on a few loci (e.g., ITS or *matK*), whole chloroplast genome data significantly enhanced node support values, particularly in resolving complex intrageneric relationships within *Murraya* (Wickett et al., 2014). However, the phylogenetic placement of certain Australian-endemic genera (e.g., *Boronia*) remains ambiguous, potentially reflecting incomplete lineage sorting caused by early rapid radiation events. These phylogenetic insights not only provide a refined framework for taxonomic revision within Rutaceae but also shed new light on the biogeographic history of the family, particularly its dispersal trajectory from Asia to Oceania. Future research should integrate nuclear genomic data to overcome the inherent limitations of single chloroplast genome analyses, especially in resolving reticulate evolution and deep phylogenetic uncertainty.

## 5 Conclusion

This comprehensive comparative analysis of 35 Rutaceae chloroplast genomes, including the newly sequenced *M. paniculata*, reveals a highly conserved quadripartite structure alongside lineage-specific variations in genome size, SSR distribution, codon usage bias, and IR boundary dynamics. The observed codon usage patterns, selection pressure signatures, and phylogenetic reconstructions collectively underscore the dual influence of purifying selection and adaptive evolution in shaping chloroplast genome architecture within the family. Notably, key photosynthetic and translational genes exhibit strong conservation, while genes such as *rpl20* and *clpP* show evidence of positive selection, indicating their potential roles in lineage-specific adaptation. The robust phylogenetic placement of *Murraya paniculata* enhances our understanding of its evolutionary relationships with economically important genera like *Citrus* and *Clausena*. Together, these findings provide valuable genomic resources and a refined molecular framework for taxonomic clarification, evolutionary studies, and biodiversity conservation in Rutaceae.

## Data Availability

The datasets presented in this study can be found in online repositories. The names of the repository/repositories and accession number(s) can be found in the article/**Supplementary Material**.

## Supplementary material

Supplementary Table 1 Summary of chloroplast genome composition and nucleotide characteristics in 35 Rutaceae species.

Supplementary Table 2 Distribution and classification of simple sequence repeats (SSRs) in Rutaceae chloroplast genomes.

Supplementary Table 3 Relative synonymous codon usage (RSCU) values of protein-coding genes across 35 Rutaceae chloroplast genomes.

Supplementary Table 4 ENC and GC3s values for chloroplast protein-coding genes in 35 Rutaceae species.

Supplementary Table 5 Results of neutrality plot analysis (GC12 vs. GC3) for chloroplast genes in 35 Rutaceae species.

Supplementary Table 6 Parity rule 2 (PR2) analysis of base composition at third codon positions in Rutaceae chloroplast genomes.

Supplementary Table 7 Coordinates of genes in correspondence analysis (COA) space based on codon usage in 35 Rutaceae Species.

Supplementary Table 8 Ka, Ks, and Ka/Ks ratios for chloroplast protein-coding genes in 35 Rutaceae species.
